# Mapping core characteristics of internet-based tools to maintain and improve population health

**DOI:** 10.1093/eurpub/ckac131.173

**Published:** 2022-10-25

**Authors:** L Maass, M Freye, CC Pan

**Affiliations:** 1 Research Center on Inequality and Social Policy, University of Bremen, Bremen, Germany; 2 Leibniz ScienceCampus Digital Public Health, Bremen, Germany; 3 Steering Committee, EUPHA-DH; 4 Institute for Information, Health & Medical Law, University of Bremen, Bremen, Germany; 5 Leibniz Institute for Prevention Research, and Epidemiology - BIPS, Bremen, Germany

## Abstract

Rapid developments and implementation of digital technologies in public health domains throughout the last decades have changed the landscape of health delivery and disease prevention globally. Many countries introduce digital interventions to their health systems to improve their populations’ health and make access to health care more accessible. Despite multiple definitions for digital public health and the development of different digital interventions, no study has analysed whether the used technologies fit the definition and the core characteristics of digital public health interventions. Digital public health for us means using digital tools to achieve public health goals. We conducted a scoping review to map the characteristics of digital public health interventions, see how the understanding of specific interventions differs between countries, and how they fit in the theoretical framework of digital public health definitions. Our review is the first to display the landscape of worldwide existing digital public health interventions that use information- and communication technologies. The study’s protocol was published in March 2022 in JMIR Research Protocols (DOI 10.2196/33404). We searched five databases (PubMed, Web of Science, CENTRAL, Ieee, and ACM) for publications. Given the broad search string, we retrieved 13,869 results screened for eligibility. A total of 1,429 publications were included for full-text screening. The study showed that the terms for specific interventions are related to the context in which they are used. Scandinavian countries displayed a different understanding of electronic health records (EHRs) than South American countries. We also identified that the separation between digital health and digital public health is blurry in praxis. Although interventions such as EHRs target individuals to improve their health, the collected data can also be pooled to allow research and the development of interventions on a public health level.

**Key messages:**

• When comparing interventions internationally, it’s best to compare based on the characteristics of the intervention rather than on the name.

• Although, in theory, the distinguishment between digital health, digital public health, and public health became more precise in recent years, the practical reality between them remains still blurry.

